# Management of Medial Femorotibial Knee Osteoarthritis in Conjunction with Anterior Cruciate Ligament Deficiency: Technical Note and Literature Review

**DOI:** 10.3390/jcm13113143

**Published:** 2024-05-27

**Authors:** Claudio Legnani, Alberto Ventura, Laura Mangiavini, Nicola Maffulli, Giuseppe M. Peretti

**Affiliations:** 1 IRCCS Istituto Ortopedico Galeazzi, Sport Traumatology and Minimally Invasive Surgery Center, 20161 Milan, Italy; 2 IRCCS Istituto Ortopedico Galeazzi, 20157 Milan, Italy; 3 Department of Biomedical Sciences for Health, University of Milan, 20122 Milan, Italy; 4 Department of Medicine, Surgery and Dentistry, University of Salerno, 84084 Baronissi, Italy; 5 Centre for Sports and Exercise Medicine, Barts and the London School of Medicine and Dentistry, Queen Mary University of London, London E1 4NS, UK; 6 School of Pharmacy and Bioengineering, Keele University Faculty of Medicine, Stoke on Trent ST4 7QB, UK

**Keywords:** knee, unicompartmental knee replacement, unicompartmental knee arthroplasty, medial osteoarthritis, anterior cruciate ligament, ACL reconstruction

## Abstract

In recent years, there has been increased interest in the management of medial femorotibial knee osteoarthritis (OA) in conjunction with anterior cruciate ligament (ACL) deficiency. Traditional treatment modalities included conservative therapy, high tibial osteotomy with or without ACL reconstruction, and total knee replacement. Since younger patients with higher physical demands are more likely to suffer from this pathological condition, reduced invasiveness, faster recovery time, and improved knee kinematics are preferred to allow for satisfying clinical and functional outcomes. Thus, a new surgical strategy combining medial unicompartmental knee replacement (UKR) and ACL reconstruction has been proposed to allow bone stock preservation, to reduce surgical morbidity and recovery time, and ultimately to improve joint kinematics and clinical outcomes. Based on the data present in the literature, in the setting of unicompartmental OA in association with ACL deficiency, UKR combined with ACL reconstruction provided encouraging early results. Studies evaluating the outcomes of combined ACL reconstruction and UKR demonstrate promising results in select patient populations. Improved knee stability, pain relief, functional recovery, and patient satisfaction improved after surgery. Moreover, the combined approach offered advantages such as reduced surgical trauma, faster rehabilitation, and preservation of native knee anatomy compared with traditional treatment strategies. However, still, high-level studies on this topic are lacking; therefore, more comparative studies reporting long-term outcomes are needed to support the potential of this combined procedure to become mainstream. In this paper, we discuss the relevant features and rationale behind the indications and technique of this combined surgical procedure, to help surgeons choose the correct therapeutic approach for a patient with concomitant medial OA and ACL insufficiency. Continued advancements in surgical techniques, patient selection criteria, and rehabilitation strategies will further enhance the success of this combined approach, offering hope to individuals with concomitant ACL injuries and unicompartmental knee OA.

## 1. Introduction

The management of medial femorotibial knee osteoarthritis (OA) in conjunction with anterior cruciate ligament (ACL) deficiency is a debatable issue and a challenge for orthopedic surgeons approaching this particular setting [1,2,3,4].

Traditional treatment modalities included conservative therapy [5], high tibial osteotomy (HTO) with or without ACL reconstruction [6,7], and total knee replacement (TKR) [1]. However, a systematic review on simultaneous HTO and ACL reconstruction demonstrated that HTO showed a higher rate of complications compared with UKR when performed in combination with ACL reconstruction [7]. In cases of severe medial cartilage damage, unicompartmental knee replacement (UKR) is a preferable bone-conserving option compared with TKR [8]. In fact, compared with UKR, TKR is usually associated with greater surgical wounds, longer operating times, a marginally increased incidence of adverse events, and decreased knee mobility. It is nevertheless a viable therapy option for some patients of osteoarthritis in the knee despite these disadvantages. Due to its advantages over TKR, such as smaller surgical incisions, quicker operating times, fewer adverse events, more knee mobility, and superior bone volume preservation, UKR has become more and more common in knee replacements in recent years. These benefits lead to better overall results and a quicker recovery from surgery. In situations with osteoarthritis in the medial compartment of the knee, UKR is especially beneficial. However, consensus exists that UKR alone is not recommended in ACL-deficient knees [3].

To avoid the drawbacks associated with TKR, a new surgical strategy combining UKR and ACL reconstruction has been proposed to allow bone stock preservation, to reduce surgical morbidity and recovery time, and ultimately to improve joint kinematics and clinical outcomes [9,10,11,12,13,14,15,16,17,18,19,20,21]. By integrating these procedures, surgeons aim to optimize knee biomechanics, enhance stability, alleviate pain, and improve overall joint function. The goal is to improve patients’ quality of life and allow this population of active subjects to return to physical and recreational activities.

Since experience in this field is still limited, this review aims to elucidate useful decision-making criteria guiding surgeons to the most appropriate therapeutic approach for the young and active patient with concomitant medial OA and ACL deficiency.

## 2. Indications and Preoperative Considerations

A consensus for the management of medial OA in conjunction with ACL deficiency is still lacking. However, with the increased number of published reports, novel evidence is rising; therefore, an individualized treatment algorithm for treating concomitant medial OA and ACL rupture should be considered. For this purpose, the distinction of the major disturbing factor between the ACL insufficiency and medial OA should be identified.

A combined approach should be considered in active patients whose primary complaint is ACL deficiency. The ACL is an essential ligament connecting the femur and the tibia. It limits the tibia’s anterior translation and rotational forces, which is important for maintaining joint stability. Sport-related injuries affecting joint ligaments in the active population may contribute to increased risks for a painful or symptomatic knee [22]. ACL incompetence causes joint instability by changing the biomechanical characteristics of the knee joint and putting the bones through excessive mechanical stress while walking. Changes in joint motion, load distribution, and stress transfer can impact joint stability and function, especially causing harm to the subchondral bone and cartilage. Usually, the anteromedial region of the tibial plateau is the most affected by cartilage degradation in medial compartment OA in ACL-intact knees. In contrast, in ACL-deficient knees, recurrent posterior femoral subluxation is caused by ACL laxity, thus leading to posteromedial wear of the tibial plateau [23]. Varus deformity, which can further change the normal biomechanics of the joint and cause higher discomfort, decreased range of motion, and the need to stop competitive or recreational sports, is more common in knees with ACL deficiencies [24]. In fact, in these cases, an untreated ACL tear may be the cause for further progression of degenerative changes due to recurrent instability leading to posterior femoral subluxation [12,25]. This surgical option may suit younger and more active patients, whose goal is to achieve a high level of activity with more stability and less pain and prevent future surgeries [26,27].

On the other hand, older and less active patients, whose primary complaint is pain from medial OA, may be candidates for TKR since additional pathologic changes like shortening of the medial collateral ligament and involvement of the lateral compartment may occur, together with pain affecting the periarticular musculature of the knee [18,28].

Since the young and active population is supposed to benefit from this combined procedure, concerns arose that improved joint function may lead to increased physical demands on the knee and subsequently increased polyethylene wear and ultimately revision surgery. Hence, patients’ compliance is critical in order to maximize the outcomes to avoid excessive implant loading and stresses on the healing graft [13].

## 3. Diagnostic Assessment

The preoperative clinical assessment comprises the patient’s history, including the presence of any concomitant ipsilateral or contralateral ligament injury and preferred sports and activity levels. As previously emphasized, the surgical approach should be tailored to patients’ expectations, and it must therefore consider the degree of recreational and sport activity level desired.

The physical examination includes the assessment of anteroposterior (AP) stability with the anterior drawer and Lachman test. In addition, collateral ligament assessment should be performed with varus and valgus stress testing at 0 and 30 degrees of knee flexion. Knee range of motion and thigh girth should also be reported.

Radiographic investigations should include weight-bearing anteroposterior and lateral views to document the degree of osteoarthritis and to check the integrity of the lateral compartment. Valgus stress radiographs may be helpful to assess the tension of the medial collateral ligament.

Magnetic resonance imaging could be useful to assess the ACL integrity, although MRI studies are not performed on a regular basis. In the presence of advanced OA, ACL-deficient knees have commonly normal examination, and the ACL status can be more reliably determined on lateral knee radiograph by determining the location of maximal tibial loss [29]. Standard post-operative X-rays can also be helpful to assess implant positioning and the presence of radiolucent lines at follow-up. Radiological outcomes can included long-leg standing radiographs to compare preoperative and postoperative leg alignment.

## 4. Authors’ Preferred Surgical Technique and Rehabilitation Protocol

**Figure 1:**
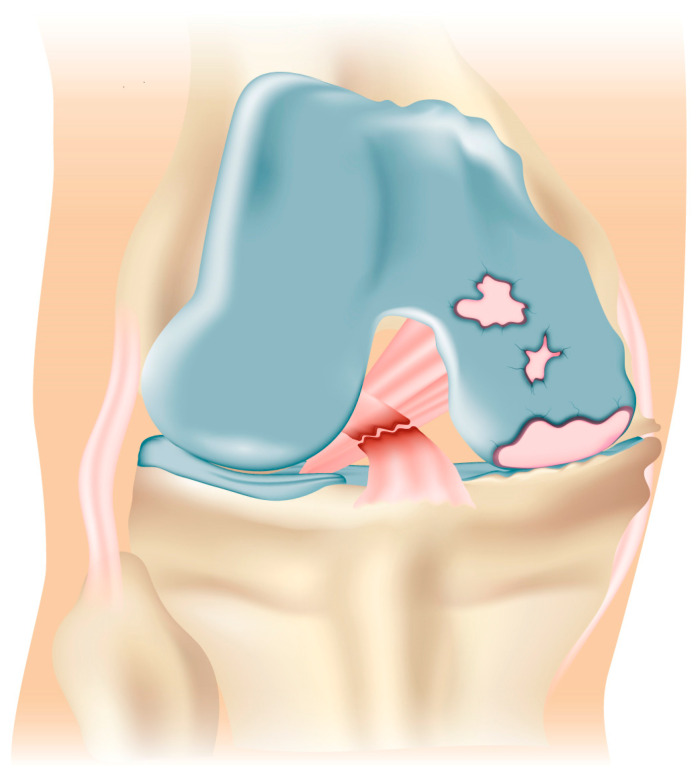
Preoperative assessment demonstrating isolated medial knee osteoarthritis secondary to ACL incompetency.

The patient is positioned supine on the operating table, with the tourniquet placed on the upper thigh and after spinal anesthesia, with a foot roll positioned to maintain 90° of knee flexion and a lateral support at the thigh. The limb to be operated is disinfected with chlorhexidine, and sterile draping is applied. The head of the fibula, lateral epicondyle, joint line, and Gerdy tubercle are among the palpated and noted landmarks. The current practice at our institution is to perform preliminary diagnostic arthroscopy. Standard anteromedial and anterolateral portals are established to assess ACL incompetency and to confirm the absence of significant degeneration of the contralateral or patellofemoral compartments (Figure 1).

**Figure 2:**
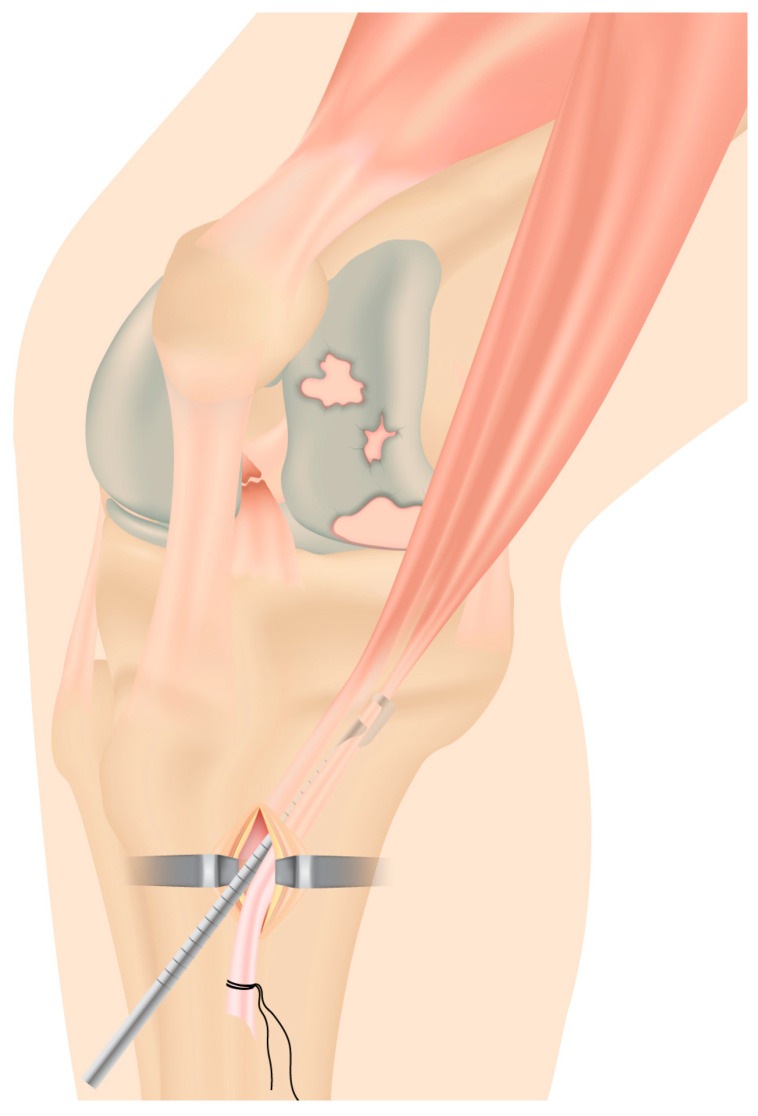
Stripping of autologous semitendinosus and gracilis tendon grafts through a minimally invasive incision over the pes anserinus.

Then, autologous semitendinosus and gracilis tendon grafts are harvested with a closed tendon stripper through a vertical incision over the pes anserinus on the anteromedial aspect of the proximal tibia and then made into a four-stranded graft. The free ends are whipstitched using no. 2 absorbable sutures (Vicryl; Ethicon, Somerville, NJ, USA) (Figure 2).

**Figure 3:**
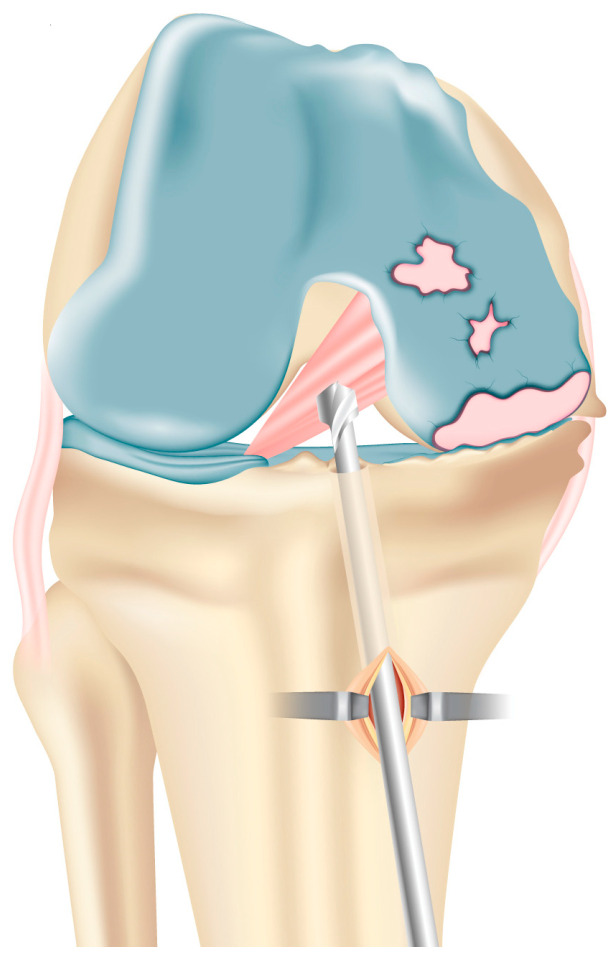
Reaming of the tibial tunnel based on graft size slightly more laterally than usual, close to the tibial tubercle. This avoids weakening of the tibial medial plateau and/or impingement on the tibial component with the late distal graft insertion.

After removing ACL remnants with the use of a motorized shaver, the anteromedial portal is used to introduce the tibial ACL guide (Acufex, Mansfield, MA, USA), which is positioned at 55 degrees slightly above the hamstring insertion. After placing it over the ACL footprint, a guidewire is put in. The tibial tunnel is reamed on the tibial plateau based on the graft size. Care is taken to exit slightly more laterally than usual, close to the tibial tubercle to ensure the tunnel is not violated by the tibial cuts for the implant of the prosthesis and to avoid the impingement on its tibial component (Figure 3).

**Figure 4:**
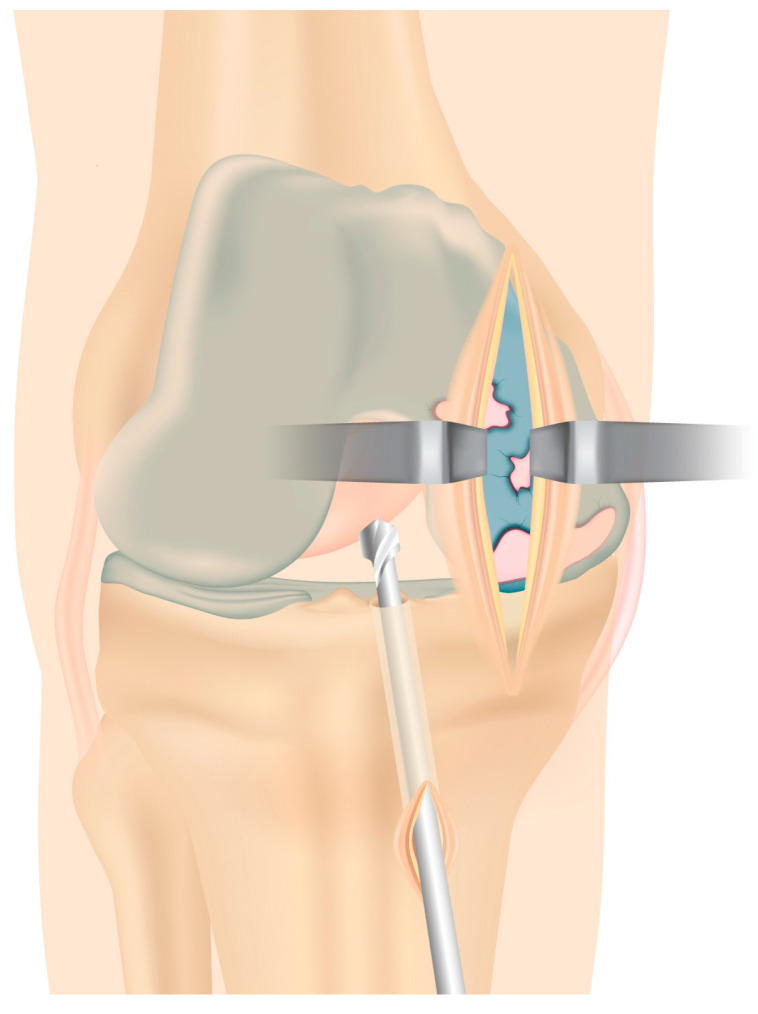
A new minimally invasive, vertical incision is performed proximally, medially to the patellar tendon to allow arthroplasty implantation.

The femoral half-tunnel is then drilled with the knee in 90 degrees of flexion as close as possible to the anatomic ACL footprint on the lateral femoral condyle with an anteromedial technique to allow the placement of the femoral tunnel in the true ACL insertion site. After the tunnel preparation, a looped no. 2 thread (Polysorb; Covidien, Mansfield, MA, USA) is delivered through the tunnels and prepared for late graft insertion. A new vertical incision is performed proximally, medially to the patellar tendon. Femoral and tibial cutting are performed with care to keep a bony bridge close to the exit of the tibial tunnel (Figure 4).

**Figure 5:**
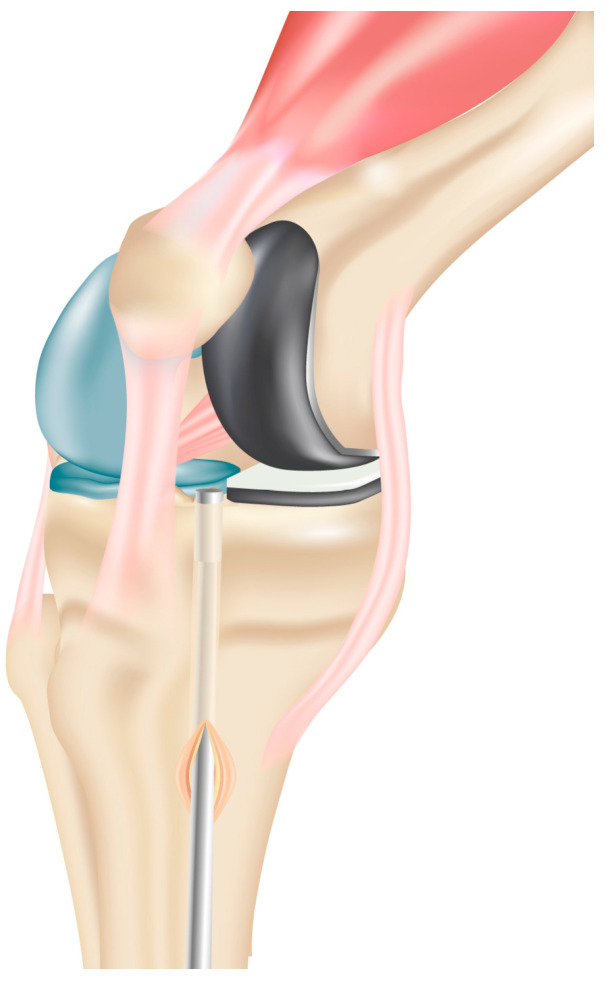
Impactor placement filling the tibial tunnel to avoid accidental penetration of the cement into the tunnel before implantation of the components.

Subsequently, the definitive femoral and tibial components of the prosthesis (Allegretto unicondylar fixed-bearing prosthesis, Zimmer Inc.; Warsaw, IN, USA) are positioned together with the fixed bearing. The final components should be cemented before the final ACL graft placement, leaving an impactor in the tibial tunnel to prevent the migration of the cement inside the tunnel (Figure 5).

**Figure 6:**
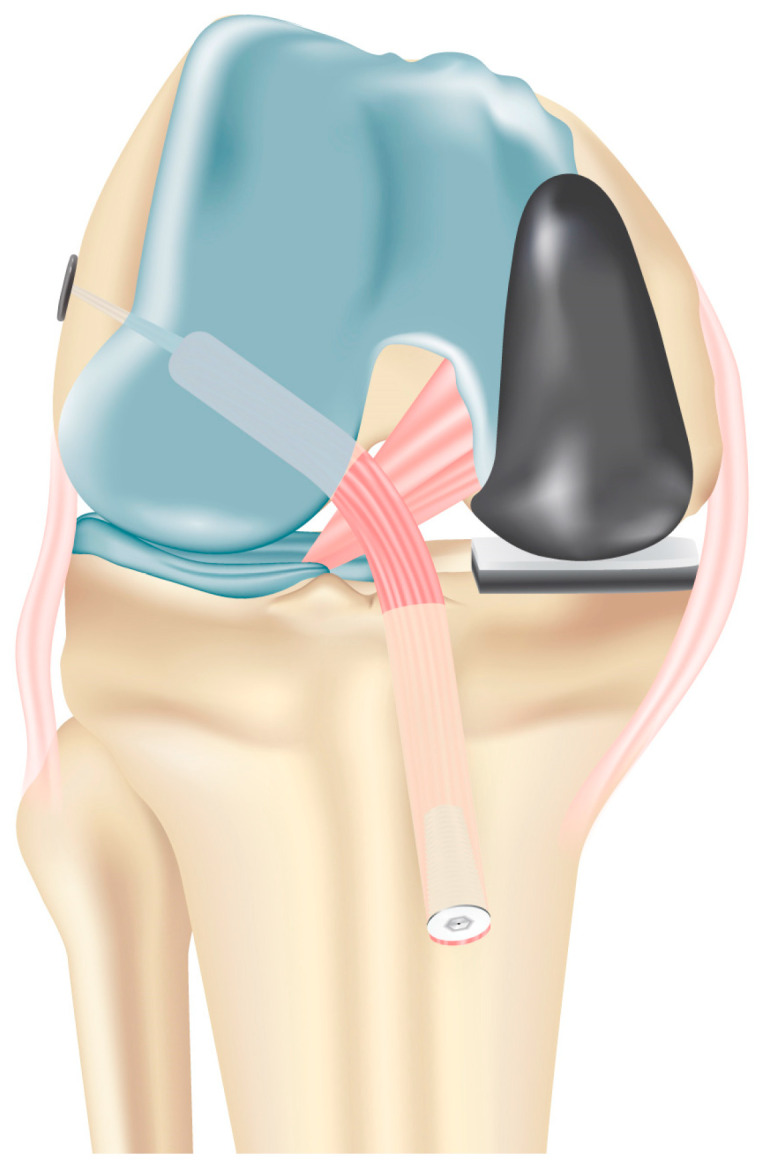
Graft fixation is performed at the end of the surgical procedure, in order to prevent the knee valgus effect on the length of the ACL. Proximal fixation is achieved with an adjustable loop button and distal fixation with a bioabsorbable interference screw.

Then, the thread is used to shuttle the graft from the tibial tunnel through the femoral tunnel through the drill holes. The senior author’s preferred proximal fixation of the neoligament is with an adjustable loop button (Tightrope; Arthrex, Naples, FL, USA). The distal fixation is achieved through a Milagro interference screw (DePuy Mitek, Raynham, MA, USA), having a diameter of 1 or 2 mm larger than the graft, introduced into the tibial tunnel with the help of a nitinol guidewire while the knee is kept at 20° of flexion under maximal manual tension. The position of the graft is checked arthroscopically to avoid intercondylar notch or tibial component impingement (Figure 6).

Hemostasis is controlled, and a drainage is positioned. The wounds are closed, and a sterile dressing applied.

A brace-free post-operative rehabilitation protocol including joint motion exercises, immediate regaining of full knee extension, and progressive weight-bearing ambulation with crutches is started the day after the operation. Partial weight-bearing is allowed for the first 3 weeks, with progression to full weight-bearing at 4 weeks. After the first month, proprioception exercises including assisted single-leg balance and heel-to-toe walking are allowed. Pearls and pitfalls when performing this intervention are summarized in Table 1.

## 5. Reported Outcomes

Based on the data present in the literature, in the setting of unicompartmental OA in association with ACL deficiency, UKR combined with ACL reconstruction provided encouraging early results [15]. Studies evaluating the outcomes of combined ACL reconstruction and UKR demonstrate promising results in select patient populations. Knee stability, pain relief, functional recovery, and patient satisfaction improved after surgery. Moreover, the combined approach offered advantages such as reduced surgical trauma, faster rehabilitation, and preservation of native knee anatomy compared with traditional TKR [21].

Preliminary reports have shown satisfying outcomes in patients treated with this combined approach [30]. Pandit et al. examined the results of combined ACL reconstruction and medial mobile-bearing UKR in a group of 15 patients aged 36 to 60 years. After an average follow-up of 2.8 years, all patients expressed high levels of satisfaction with increased occupational and physical activity [12]. Krishnan et al. reported similar excellent clinical outcomes after an average follow-up of two years in nine patients. No revision was required, and the Knee Society score (KSS) was 196 points, while the Oxford knee score was 11 [11]. No patients reported knee instability. Tinius et al. reported a significant improvement of KSS from 77.1 to 166.0 after an average follow-up time of 53 months in 27 patients undergoing combined ACL and fixed-bearing medial UKR [13]. Similarly, Krishnan et al. and Dervin et al. reported good to excellent results in 9 and 10 patients, respectively, at an average follow-up of 2 years (KSS = 196 points, Oxford knee score (OKS) = 11) [10,11]. More recently, Weston-Simons et al. followed up 51 patients after the implantation of an Oxford knee prosthesis combined with ACL reconstruction for an average follow-up of 60 months (range from 12 to 120 months) and reported statistically significant improvements in functional and subjective scores from preoperative to postoperative status [9]. Tian et al. also using a mobile-bearing prosthesis evaluated KSS, OKS, and KSS after an average follow-up time of 52 months from surgery; most of the 28 patients reported good or excellent satisfaction with the procedure [18].

On the other hand, a high complication rate with a significant percentage of complications requiring further surgery (25%) was reported by Iriberri et al., although the sample size (eight patients) was quite limited [20].

Aslan et al. examined 12 patients after an average follow-up of 45.6 months after simultaneous mobile-bearing UKR and ACL reconstruction and reported significant improvements in terms of the OKS, EQ-5D-3L, and EQ-visual analog scale (VAS). No complications were reported at follow-up [31].

According to Foissey et al., 10 patients underwent robotically assisted UKR in addition to hamstring ACL surgery after an average follow-up of 45 months. The patients’ mean International Knee postoperative function score was 93, and their average Tegner score was 4.5 [32]. Jaber et al. followed up 23 patients who had undergone mobile-bearing UKR in addition to ACL repair for ten years. The average Lysholm score was 85.5, while the average OKS was 40. None of the patients had knee instability, and they were all able to return to their physical and sports routines. The authors reported a survival rate of 91.4% at 14.5 years [33].

In the study by Kurien et al., 24 patients who underwent simultaneous single-stage ACL reconstruction and fixed bearing medial UKR were evaluated after a mean follow-up of 5.1 years. The average Lysholm score was 92, and the average OKS score was 46. Fixed-bearing UKR was chosen in order to reduce the possibility of bearing dislocation [34].

In the case series previously reported by the authors, 12 patients with primary ACL lesion and concomitant medial compartment symptomatic OA treated from 2006 to 2010 were followed up for an average time of 7.8 (range 6 to 10) years. The mean overall KOOS score, the OKS, the WOMAC index, and the KSS increased from preoperative status; in addition, the authors reported no clinical evidence of instability in any of the knees as evaluated with clinical and instrumented laxity testing [17]. When compared with TKR, simultaneous UKR and ACL reconstruction demonstrated clinical and radiographic results comparable to TKR 10 years after surgery with no increased risk of complications [35].

Concerning the type of prosthesis implanted, there has been no reported difference in clinical outcomes between fixed-bearing and mobile-bearing UKRs or in implant wear. Proper tensioning of the collateral ligaments or ACL is crucial for successful mobile-bearing UKRs, which rely on the integrity of both the ACL and the medial collateral ligament. Using mobile-bearing UKRs can make it challenging to achieve proper ligament balancing during the combined procedure as graft fixation occurs after the prosthesis is implanted. Therefore, to perform combined UKR and ACL reconstruction, the fixed-bearing prosthesis is the authors’ favorite implant as we recommend using a prosthesis that does not rely on ligament tension when implanting its components.

A summary of previous studies is reported in Table 2.

## 6. Return to Sports

Returning to sports and physical activities is an important element for individuals who have had knee reconstructive surgery and constitutes a challenge for surgeons [36]. The postoperative period following combined UKR and ACL restoration is more difficult in comparison with UKR alone since patients have a lengthier recovery time, and resuming an active lifestyle may require more than 6 months after surgery. Therefore, patient compliance is crucial to the successful management of the perioperative and rehabilitation period in a larger time frame, to enhance patients’ outcomes and minimize complications. Furthermore, younger age (55 years or under) has been associated with a higher risk of early prosthesis failure, perhaps because these patients are more active, have high expectations about the capability of resuming sport and recreational activities, and could be potentially not satisfied also in the presence of successful outcomes. Therefore, caution should be utilized in this population. In order to prevent premature implant failure due to wear, surgeons frequently advise patients to refrain from highly demanding activities, privileging low-impact sports such as swimming, cycling, hiking, or fitness training. There is a worry that improved joint function may result in significant physical demands on the knee, even when patients are instructed to limit their activity, and the limited implant duration represent another significant issue in this cohort of patients.

## 7. Complications

The combined procedure is proven to be a safe and effective technique with implant survivorship ranging from 87.5% to 100%. According to the current literature, few complications were noted. In one patient, an undisplaced fracture of the anterior cortex of the medial tibial plateau was reported, while an iatrogenic grade 2 injury to the medial collateral ligament treated conservatively was observed in another subject [34]. Post-operative stiffness requiring arthroscopic arthrolysis occurred two and three months after surgery in two patients, respectively; in both cases, the patient had fully recovered their flexion at the last follow-up [32]. Infrequent prosthetic failures requiring revision were related to implant infection or symptomatic lateral osteoarthritis progression [9,14,16,17,20]. Inlay dislocation has been reported in three patients who underwent mobile bearing implant placement [9,18]. The use of fixed-bearing prostheses may limit this risk and allows optimal graft tension since their technical features do not rely on natural tension in the ligaments while implanting the components [14,17]. However, given its complexity, this combined procedure must be approached with caution by well-experienced surgeons, familiar with both arthroplasty and ligament reconstruction.

## 8. Conclusions

The increased demand from younger patients with higher physical demands with medial femorotibial knee OA in conjunction with ACL deficiency has led to a new surgical strategy combining medial UKR and ACL reconstruction.

This technique, aiming to reduce invasiveness and recovery time and to improve knee kinematics, has led to satisfying clinical and functional outcomes, with a high rate of implant survivorship and few complications reported.

In order to achieve optimal results, patient compliance and preoperative planning are crucial, and several technical features have to be considered to succeed while performing this technically demanding surgery.

Still, high-level studies on this topic are lacking; therefore, more comparative studies reporting long-term outcomes are needed to support the potential of this combined procedure to become conventional.

## 9. Future Directions

The combined ACL reconstruction and UKR represent a comprehensive approach to addressing complex knee pathologies. By integrating ACL reconstruction and UKR, surgeons can effectively restore knee stability, alleviate pain, and improve functional outcomes in select patient populations. Continued advancements in surgical techniques, patient selection criteria, and rehabilitation strategies will further enhance the success of this combined approach, offering hope to individuals with concomitant ACL injuries and unicompartmental knee OA. Further comprehensive follow-up studies with large sample sizes are necessary, given the significance of assessing the long-term outcomes and potential problems associated with these procedures. Such studies will make a substantial contribution to the continuing evaluation of the long-term safety and effectiveness of combined UKR and ACL reconstruction.

## Figures and Tables

**Table 1:** Pearls and pitfalls when performing combined medial UKR and ACL reconstruction.

Pearls	Pitfalls
Drilling the tibial tunnel slightly more laterally as usual close to the tibial tubercle avoids weakening of the tibial medial plateau and/or impingement on the tibial component with the distal graft insertion.	Technically demanding procedure, with risks of potential graft impingement, undersizing of the tibial component and postoperative stiffness.
After tunnel preparation, keep a flexible wire as a guide for late graft insertion.	It is important to ensure that the graft is fixed at the end of the surgical procedure because of the knee valgus effect on the length of the ACL.
Before implantation of the components, the tibial tunnel should be filled with an impactor to avoid accidental penetration of the cement into the tunnel.	The use of a fixed-bearing prosthesis allows for optimal graft tension and limits the risks of inlay dislocation.
A combined procedure in one session allows shorter hospitalization and reduced costs without the need for two surgical procedures.	To avoid the theoretical role of a stress riser by the drill hole, the tibial tunnel should be drilled more vertically as usual to prevent the risk of proximal tibia fracture.

UKR: unicompartmental knee replacement; ACL: anterior cruciate ligament.

**Table 2:** Summary of all available studies on combined UKR and ACL reconstruction.

Author	Year	No. of Patients	Mean Age	Mean Follow-Up	Post-Op Mean Outcome Score	Complications (Rate)
Pandit et al. [12]	2006	15	49.8	2.8 years	OKS: 46 KSS objective: 99 KSS functional: 96 Tegner activity level: 3.8	One (6.7%) infection and two-stage revision to a TKR
Krishnan et al. [11]	2009	9	56	24 months	WOMAC: 24 OKS: 11 KSS:196	None reported
Tinius et al. [13]	2012	27	44	50 months	OKS: 166 KSS objective: 83.2 KSS functional: 82.7	None reported
Weston-Simons et al. [9]	2012	51	51	60 months	OKS: 41 AKS functional score: 95 AKS objective score: 75 Tegner activity level: 3.5	One (2%) infection and two-stage revision to a TKR One (2%) bearing dislocation One (2%) symptomatic lateral osteoarthritis and conversion to TKR
Tian et al. [18]	2016	28	50.5	52 months	OKS: 43 KSS objective: 84.5 KSS functional: 86.9 Tegner activity level: 5.3	Two (7%) bearing dislocations
Iriberri et al. [20]	2018	8	52	175 months	KSS: 154 WOMAC: 26 VAS: 3	One (12.5%) symptomatic lateral osteoarthritis and conversion to TKR One (12.5%) external meniscus tear repair
Tecame et al. [19]	2019	24	47.8 48.4	53 months 42 months	WOMAC: 79.3 mobile, 81.3 fixed KSS functional: 86.2 mobile, 84.7 fixed KSS objective: 73.4 mobile, 77.3 fixed	None reported
Kennedy et al. [16]	2019	75	52.6	6.4 years	OKS: 41 Tegner activity level: 3.6	Three (3.9%) revisions to TKR
Ventura et al. [17]	2020	12	54	7.8 years	KOOS: 80.2 OKS: 42.4 WOMAC: 84.9 AKS objective score: 75 AKS functional score: 88	One (8.3%) symptomatic lateral osteoarthritis and conversion to TKR
Aslan et al. [31]	2022	12	Not reported	45.6 months	OKS: 45.2	None reported
Kurien et al. [34]	2022	24	48.8	5.1 years	Lysholm: 92 OKS: 46 Tegner activity level: 3.96 VAS: 0	One (4.1%) undisplaced anterior cortex fracture of the medial tibial plateau and one (4.1%) iatrogenic grade 2 injury to the medial collateral ligament treated conservatively
Jaber et al. [33]	2023	23	48	10 years	Lysholm: 85.5 OKS: 40 Tegner activity level: 3.6 VAS: 1.3 UCLA activity level: 6.7 AKS objective score: 91.5 AKS functional score: 90	Two (8.6%) revisions with conversion to total knee arthroplasty at 6 and 12 years postoperatively
Foissey et al. [32]	2023	10	57	45 months	IKS knee score: 96 IKS function score: 93 Tegner activity level: 4.5	Two (20%) reoperations due to postoperative stiffness

## Data Availability

Raw data will be provided on request.
